# Maternal and fetal health in the digital twin era

**DOI:** 10.3389/fped.2023.1251427

**Published:** 2023-10-12

**Authors:** Valeria Calcaterra, Valter Pagani, Gianvincenzo Zuccotti

**Affiliations:** ^1^ Department of Internal Medicine and Therapeutics, University of Pavia, Pavia, Italy; ^2^ Pediatric Department, Buzzi Children’s Hospital, Milano, Italy; ^3^ Grant & Research Department-LJA-2021, Asomi College of Sciences, Marsa, Malta; ^4^ Department of Biomedical and Clinical Science, University of Milano, Milano, Italy

**Keywords:** 1,000 days, children, digital twin era, origins of health, chronic disease, maternal, fetal

## 1. Introduction

Maternal and neonatal mortality continue to be significant global concerns, with a woman or newborn dying every 7 s during pregnancy, childbirth, or the early postnatal period (1). The first 1,000 days of life, which encompasses pregnancy and the early years, is a crucial period for lifelong health (2–4). During this time, there is an increased risk of developing non-communicable diseases (NCDs) such as diabetes, cardiovascular diseases, cancer, and chronic respiratory diseases. In fact, the Developmental Origins of Health and Disease hypothesis suggests that environmental conditions during fetal and early postnatal development have lasting effects on growth, structure, and metabolism, influencing long-term health and well-being (2–4).

Using machine learning by identifying key risk factors of NCDs over the early lifespan, a predictive disease model and a digital twin system (DTs) will be created.

The current literature for DTs applications in healthcare is relatively new compared to other fields. DTs has been reported in various area of precision medicine including management of asthma (5), diabetes (6, 7), precision cancer care (8), personalized cardiovascular system model (9), predictive simulations of treatment response in infectious diseases (10, 11). Eventhough, there are many challenges and barriers that must be overcome before a DTs can be used in health care (12), DTs may have an increasing future use and will become a new platform for personal health management and healthcare services (13).

The use of DTs in the setting of maternal and fetal medicine may represent a perspective approach to protect maternal and fetal health and to reduce the burden of diseases. Sharing the opinions has the power to make an opportunity to debate on the topic and to explore the potential of DTs.

## 2. Maternal and fetal health in the digital twin era

To address the challenges of the NCDs, monitoring the health and growth of babies from conception to age two has been recognized as a “golden window of opportunity” for reducing neonatal morbidity and mortality and promoting individual health (2–4).

Currently, gynecological obstetrical ultrasounds are routinely used in prenatal care to monitor the fetal growth and development, and identify potential health concerns (14). However, interpreting the data from these ultrasounds can be difficult, prone to human error, and often fails to integrate various factors that impact pregnancy outcomes and the child's growth trajectory, such as the maternal gestational status, paternal condition, lifestyle behaviors, and gene-environment interactions.

In the era of DTs advanced technologies including ultrasound imaging, data analytics, and artificial intelligence (AI) are revolutionizing the monitoring and care of pregnant women and fetus. DTs have gained attention in the medical field in recent years (13). A DT consists of a physical entity in the real world, a digital representation in software form, and the data that connects these two elements (15, 16). Using DTs have been used to create virtual representations of organs, systems, and even entire patients, aiding in diagnosis and treatment. Although the use of DTs in perinatal care is a relatively new field, it holds tremendous promise (17).

Studies have shown that using DTs leads to more accurate monitoring of fetal growth and development, especially in managing high-risk pregnancies (17). DTs also enhance the detection of fetal anomalies, reducing the need for additional diagnostic procedures. Development of digital fetal heart models has been reported for the prenatal diagnosis of congenital heart diseases []. Furthermore, DTs enable more accurate predictions of fetal heart rate variability and monitoring of fetal brain development (17–19). As reported, a dynamic DTs represents a useful strategy for prevention, diagnosis, therapy and prediction of disease during the life in a individual who experienced hypertension during periconception, optimizing children health care (12). Recently an integration between DTs and fetal circulatory models has been also proposed to develop and implement the perinatal life support system (20).

**Figure 1:**
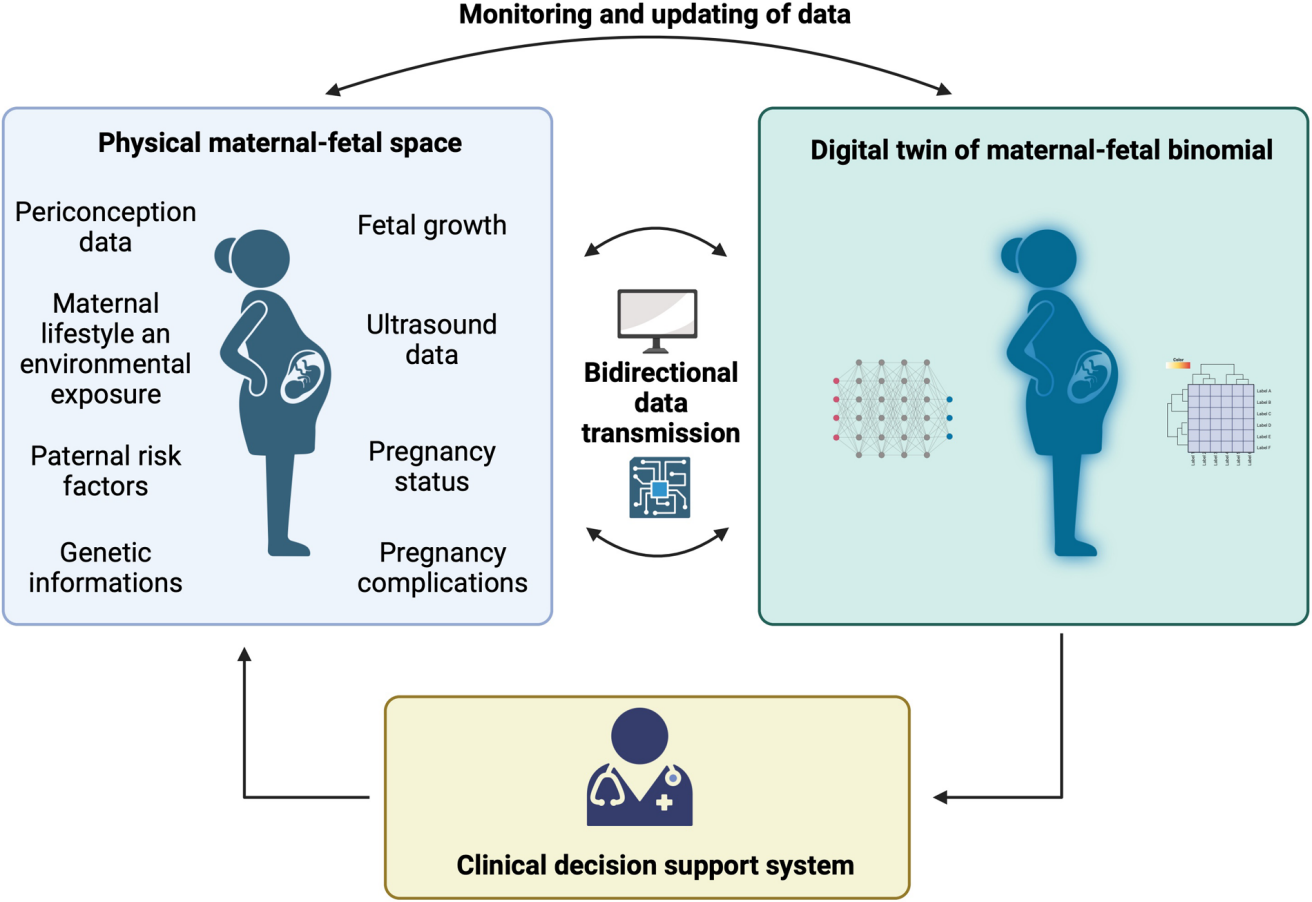
Scheme of a maternal and fetal digital twin system. Maternal and fetal data collection is performed and the informations are transformed into digital data, to adapt care by a health care professional. Digital twin system enables the continuous connection between the physical and digital space to monitor and update existing records.

By combining state-of-the-art ultrasound machines, cutting-edge image processing algorithms, and sophisticated modeling techniques, highly detailed and precise virtual representations of the fetus can be generated. The DT system can incorporate data on maternal and paternal risk factors, as well as environmental factors, to assess the overall risk to the newborn. Real-time data collection and analysis through the DT system enable early detection of maternal and fetal health risks, empowering healthcare providers to intervene proactively and make informed decisions. This approach supports personalized care plans, facilitates shared decision-making with expectant parents, and enhances patient engagement. In Figure 1, a scheme of maternal-fetal DTs is reported.

The DT provides healthcare professionals with an immersive and dynamic platform for monitoring the well-being of both mother and baby throughout pregnancy, childbirth, and the postnatal period. DT can dynamically reflect the changes in the maternal-fetal binomial. As reported AI uses and algorithm has been reported to predict fetal lung maturity (21), perinatal outcome (22), brain damage (23), estimate gestational age in late pregnancy (24) and classify standard fetal brain images as normal or abnormal offering (25). Combining prenatal information with longitudinal clinical, immunological, biochemical, behavioral, and omics profiles in the early postnatal period can create a digital replica, enabling the implementation of personalized programs and revolutionizing disease management (14, 15).

Additionally, DTs can be utilized to gain insights through simulations (14, 15). By leveraging machine learning techniques to identify key risk factors throughout an individual's life, we can adopt the training of a foundational predictive model.

This approach enables the creation of predictive models that can anticipate the likelihood of developing diseases (15). The integration of vast amounts of data, the formulation of algorithms, and the establishment of risk scores for predicting susceptible pediatric populations all play a crucial role in the realm of public health. Moreover, this goes beyond advancing our scientific understanding of disease mechanisms, as it also enhances the potential for targeted therapeutic interventions.

## 3. Discussion

Accurate predictions of both normal and pathological states in patients rely on a comprehensive understanding of how genes and the environment interact, both at an individual and population level. The emerging field of systems medicine embraces a holistic and integrative approach to patient care. By introducing digital and technological advancements during the first 1,000 days of life, we can offer an innovative perspective that improves various aspects of the diagnostic process. This approach includes constructing predictive models, obtaining indicators of biological processes and pharmacological responses to therapeutic interventions, and ultimately reducing the burden of morbidity and early mortality.
